# Differential Expressions Between Colostrum and Mature Milk in Primiparous Holstein Cows with Integrations of Lipidomic and Proteomics

**DOI:** 10.3390/foods15183184

**Published:** 2026-09-09

**Authors:** Hao Wu, Peng Wang, Pengyun Ji, Yukun Song, Xiangshu Piao, Lu Zhang, Bingyuan Wang, Guoshi Liu

**Affiliations:** 1 National Engineering Laboratory for Animal Breeding, Key Laboratory of Animal Genetics and Breeding of the Ministry of Agricultural, Beijing Key Laboratory for Animal Genetic Improvement, College of Animal Science and Technology, China Agricultural University, Beijing 100193, China; 18800160525@163.com (H.W.); wangpeng2105@163.com (P.W.); luzhang2018@cau.edu.cn (L.Z.); 2 Beijing Jingwa Agricultural Science and Technology Innovation Center, Beijing 101206, China

**Keywords:** primiparous Holstein cows, lipidomics, proteomics, integrated analysis, α-ketoglutarate

## Abstract

In the current study, by integrating the analyses of lipidomics and proteomics, the compositional differences between colostrum and mature milk of Holstein cows were systematically investigated. We have identified a total of 1863 lipid metabolites and 1690 proteins. A small portion of them were highly differentiated between colostrum and mature milk. Among them, 23 highly differentiated lipid metabolites and 17 proteins were screened to run correlation analysis. The results showed that they are highly correlated; therefore, they could serve as biomarkers to distinguish colostrum from mature milk. Furthermore, among lipid metabolites, triacylglycerol (TG) (10:0_11:0_12:0) and sphingomyelin (SM) (20:0;2O/44:11) were identified as key regulatory molecules in glycerophospholipid metabolism and sphingomyelin metabolism. In proteins, cadherin 3 (CDH3) protein and alpha-S1-casein (CSN1S1) protein have been identified as potentially important characteristic proteins. In addition, the level of α-ketoglutarate (α-KG) in colostrum was two-fold lower than that in mature milk and its level strongly correlated with several differentiated metabolites and tricarboxylic acid (TCA) cycle-related proteins, including isocitrate dehydrogenase 1 (IDH1), aconitase 1 (ACO1), and dihydrolipoamide dehydrogenase (DLD). This indicates that α-KG is one of the most important differential molecules to determine the compositions of colostrum and mature milk. These findings provide molecular basis for understanding the nutritional value and the regulatory mechanisms related to the compositions of colostrum and mature milk.

## 1. Introduction

Milk is a nutrient-rich food with high essential proteins, fats, and calcium [1]. It has a vast consumer market. From farm to commercial milk, several processes are required, but the nutritional values remain a key determinant of milk’s quality [2]. The colostrum, secreted within 72 h after calving, is rich in various bioactive components with a variety of important functions, including enhancing immune responses, promoting growth and development, and regulating gastrointestinal function. As a result, colostrum can be used as a raw material for the development of functional foods, nutritional supplements, and sometimes, medicines [3]. Lipids and proteins are important components in milk, featuring diversity and complexity [4]. The compositional differences in lipids and proteins between colostrum milk and mature milk represent a crucial physiological adaptation by the maternal body to match the nutritional requirements of newborns during the lactation period [5,6]. Therefore, a systematic study of the synthetic mechanisms of milk lipid and protein at different lactation stages has a great scientific and practical significance for improving the quality assessment of dairy products and achieving precise nutritional supply.

Even though there are a large number of reports on the bovine milk component analyses, they are based on traditional methods including the Kjeldahl method [7], the Babcock method [8], and enzymatic methods [9]. These methods all limit to detect a unique substance and the results are easily interfered by various factors with the accurate shortcomings. As the rapid development of modern molecular techniques, omics technologies, including genomics [9], lipidomics [10], and proteomics [11], have been widely applied in many fields such as medicine, food science and nutritional metabolism. For example, lipidomics enables qualitative and quantitative analyses of diverse fat metabolites with throughput and high sensitivity [12]. Quantitative proteomics can detect and quantify thousands of proteins in the tissues of organisms, providing a key technical means for understanding the composition and function of proteins in organisms [13].

Previous studies have conducted metabolomic analyses of human milk and the results showed that the levels of valine, leucine, betaine, and creatinine were significantly higher in colostrum than mature lactation milk, whereas glutamic acid, caprylic acid, and capric acid were significantly higher in mature milk than in colostrum [14]. In different species, proteomic analysis using data-independent acquisition (DIA) technology identified β-2-glycoprotein 1 and aminopeptidase as potential biomarkers of goat milk, while angiopoietin-1 and serpin G1 were identified as potential biomarkers of cow milk [15]. Comparative proteomic analyses of the whey proteins and milk fat globule membrane (MFGM) proteins between Guanzhong dairy goats and Holstein cows showed that the most enriched pathways were metabolic pathways in goats and disease-related pathways in cows [16]. Recently, integrated lipidomic and proteomic approaches have been applied to human, porcine, bovine, buffalo, and yak milk. And they reveal significant interspecies differences in metabolites, lipids, and proteins, with bovine milk exhibiting higher concentrations of iminodibenzyl and osteopontin (OPN) than the other species [17]. However, although many studies have conducted comprehensive analyses of the components of human milk and goat milk, especially those of multiparous dairy cows and their products, there are relatively few studies on the composition of milk from primiparous dairy cows due to the differences in lactation stage and parity.

In this study, lipidomic and proteomic analyses were performed to characterize the compositional differences between colostrum and mature milk of primiparous Holstein cows. The objective is to decipher the distinct and interconnected lipidomic and proteomic profiles of colostrum versus mature milk in primiparous Holstein cows to identify specific quality biomarkers. We hypothesized that the transition from colostrum to mature milk would be accompanied by coordinated changes in lipid metabolites and proteins, including stage-specific lipid classes, protein biomarkers, and alpha-KG-associated metabolic signature.

## 2. Materials and Methods

### 2.1. Milk Sample Collection

From December 2024 to January 2025, a cohort of eight healthy primiparous Holstein cows was randomly selected for monitoring at the Dairy Demonstration Farm of the Beijing Jingwa Agricultural Technology Innovation Center in Pinggu District, Beijing, China. Colostrum samples (Group C) were collected within 2 h postpartum as the first lactation yield, and mature milk samples (Group M) were collected approximately 15 days after parturition. All experimental cows were first-calving heifers with normal delivery and no metabolic disorders (such as ketosis or postpartum hypocalcemia), mastitis, or reproductive system diseases. The body condition score (BCS) of the cows ranged from 3.0 to 3.5, with a mean age of 27.56 months. Milk samples were obtained during routine milking and gently mixed immediately after collection to ensure homogeneity. Samples were transported to the laboratory at 4 °C, aliquoted into sterile centrifuge tubes, and stored at −80 °C without the addition of any preservatives. Throughout the study, the cows were housed in free-stall barns under standardized management conditions. Each cow was provided with an individual stall to avoid overcrowding. All animals had ad libitum access to water and were fed a total mixed ration (TMR; during the prepartum period, the diet consisted of 49.80% corn silage, 21.05% oat forage, 16.19% prepartum concentrate supplement, and 12.96% water; during the postpartum lactation period, the diet consisted of 53.70% corn silage, 6.59% alfalfa forage (RFV > 170), 2.44% oat forage, 28.07% lactating-cow concentrate supplement, 0.66% fat powder, and 8.54% water) following routine farm-management practices. Ambient temperature varied naturally with seasonal changes during the trial. Management and environmental conditions were consistent across groups to minimize potential confounding factors. The study protocol was approved by the Ethics Committee of China Agricultural University (Approval No. AW21605202-1-15).

### 2.2. Metabolite Extraction and LC–MS/MS Analysis

#### 2.2.1. Metabolite Extraction

A 100 μL of milk sample was transferred to a 1.5 mL Eppendorf tube, followed by the addition of 480 μL extraction solvent (MTBE: MeOH = 5:1, *v*/*v*) containing isotope-labeled internal standards. The mixture was vortexed for 30 s, sonicated in an ice-water bath for 10 min, and then incubated at −40 °C for 1 h. Samples were centrifuged at 3000 rpm (RCF = 900× *g*) for 15 min at 4 °C. A 350 μL aliquot of the supernatant was collected and evaporated to dryness in a vacuum concentrator. The dried residue was reconstituted in 100 μL of reconstitution solvent (DCM: MeOH = 1:1, *v*/*v*), vortexed for 30 s, and sonicated again in an ice-water bath for 10 min. After centrifugation at 12,000 rpm (RCF = 13,800× *g*) for 15 min at 4 °C, 75 μL of the supernatant was transferred into LC vials for instrumental analysis. For quality control (QC), equal aliquots of supernatants from all samples were pooled and analyzed under the same conditions.

#### 2.2.2. LC-MS/MS Analysis

Chromatographic separation was performed using a Vanquish™ UHPLC system (Thermo Fisher Scientific, Sunnyvale, CA, USA) equipped with a Phenomenex Kinetex C18 column (2.1 mm × 100 mm, 2.6 μm). Mobile phase A consisted of water–acetonitrile (40:60, *v*/*v*) with 10 mmol/L ammonium formate, and mobile phase B consisted of acetonitrile–isopropanol (10:90, *v*/*v*) with 10 mmol/L ammonium formate. The injection volume was set to 2 μL. Mass spectrometric detection was conducted on an Orbitrap Exploris 120™ (Thermo Fisher Scientific, Sunnyvale, CA, USA) under the control of Xcalibur software (v4.4, Thermo) in both positive and negative electrospray ionization modes.

The key MS parameters were as follows: sheath gas flow rate, 30 Arb; auxiliary gas flow rate, 10 Arb; capillary temperature, 320 °C (both positive and negative modes); full MS resolution, 60,000; MS/MS resolution, 15,000; normalized collision energy (NCE), 15/30/45; spray voltage, 3.8 kV (positive mode) or −3.4 kV (negative mode).

#### 2.2.3. Data Processing

Raw data files were converted to mzXML format using ProteoWizard (3.0.24254 64-bit). Retention time correction, peak detection, peak extraction, peak integration, and peak alignment were performed using the XCMS package with the parameters set to minfrac = 0.5 and cutoff = 0.3. Lipid identification was conducted using the XCMS package in R software (version 3.6.1, R Core Team, Auckland, New Zealand), and the LipidBlast database [18].

### 2.3. Lipidomics Data Analysis

#### 2.3.1. Raw Data Preprocessing

The raw dataset contained three QC samples and 16 experimental samples, from which 26,705 features were extracted. Data preprocessing involved the following steps: (1) Outlier filtering: noise peaks were removed based on the relative standard deviation (RSD, coefficient of variation, CV) of each feature. (2) Missing value filtering: features with a missing rate >50% across the 16 experimental samples were excluded. This threshold was selected to retain features detected in at least half of the experimental samples while reducing the loss of potentially relevant features due to sporadic missing values. (3) Missing value imputation: missing values were imputed using half of the minimum detectable value of the corresponding feature. (4) Normalization: peak areas were normalized to internal standards. After preprocessing, 18,898 features were retained for further analysis.

#### 2.3.2. Statistical Analysis

After preprocessing, metabolite classification and normalization were performed. Paired *t*-test analysis was conducted using R (version 3.6.3). For univariate analyses and quantitative comparisons, data are presented as mean ± standard deviation (SD). For multivariate analysis, the data were log-transformed and centered (CTR) for principal component analysis (PCA) and log-transformed and UV-scaled for orthogonal partial least squares discriminant analysis (OPLS-DA) using SIMCA software (v18.0.1, Sartorius Stedim Data Analytics AB, Umea, Sweden). Differential metabolites were screened based on variable importance in projection (VIP > 1.0), *p* < 0.05 (Paired *t*-test), and fold change (FC < 0.5 or >2). Visualization and pathway analysis were conducted as follows: volcano and stick plots were generated using R (ggplot2, v3.3.5); hierarchical clustering heatmaps using R (pheatmap, v1.0.12); correlation heatmaps using R (corrplot, v0.89); and KEGG pathway bubble plots using R (KEGGgraph, ggplot2, v1.46.0, v3.3.5). For correlation analyses, Spearman correlation was used to assess associations between differential metabolites and proteins, whereas Pearson correlation was used specifically for the nine-quadrant association analysis.

### 2.4. Proteomics Sample Preparation and Mass Spectrometry Analysis

#### 2.4.1. Protein Extraction

A 500 μL aliquot of bovine milk was centrifuged at 12,000 rpm for 10 min, and the supernatant was transferred to a new tube. Five volumes of prechilled acetone were added, and proteins were precipitated overnight at 4 °C. The sample was centrifuged at 12,000 rpm for 10 min, and the protein pellet was collected. The pellet was resuspended in 300 μL of RIPA lysis buffer and sonicated in an ice bath for 20 min. After centrifugation at 12,000 rpm for 10 min, the supernatant was collected for further analysis.

#### 2.4.2. BCA Protein Quantification

Protein concentration was determined using the bicinchoninic acid (BCA) assay. Briefly, 200 μL of BCA working reagent was added to each well of a 96-well plate. A series of seven BSA standard dilutions and one blank control were prepared. Each well received 20 μL of diluted sample or standard solution. Plates were incubated at 37 °C with gentle shaking for 30 min, and absorbance was measured at 562 nm. Protein concentrations were calculated from the standard curve.

#### 2.4.3. Reduction, Alkylation, and Enzymatic Digestion

A total of 30 μg (60 μL) of protein solution was transferred to the reaction plate. Reagents were sequentially added according to the instructions of the kit (NO: OSFP0001-AUTO, Shanghai Yicang Biotechnology Co., Ltd. Shanghai, China), add the reagents in sequence: reagent H, followed by a water/reagent G mixture. The mixture was vortexed at 800 rpm for 1 min, magnetically separated for 1 min, and the supernatant was discarded. Reagent A was added to the sample, vortex-mixed for 1 min, and incubated at 95 °C for 5 min at 1200 rpm. After cooling to room temperature, reagent I was added, followed by incubation at 37 °C for 10 min at 1200 rpm. The supernatant was discarded, and the sample was washed twice with reagent G at 37 °C. Subsequently, reagent B and reagent J were mixed and transferred to the reaction plate for incubation at 37 °C for 2.5 h at 1200 rpm. Reagent C was added and incubated at 37 °C for 1 min at 1200 rpm. The mixture was desalted on a desalting plate by sequentially applying reagent I, reagent E, the digested peptide solution, reagent D, and finally reagent F under positive pressure. The eluate was vacuum-dried and stored for subsequent reconstitution and LC–MS/MS analysis.

#### 2.4.4. nanoLC–MS/MS Analysis

Approximately 200 ng of total peptides from each sample were separated on a nanoUPLC system (nanoElute 2, Bruker, Germany) and analyzed on a timsTOF Pro 2 mass spectrometer (Bruker, Germany) equipped with a nano-electrospray ion source. Separation was performed on a reversed-phase column (PePSep C18, 1.9 μm, 75 μm × 15 cm; Bruker, Germany). Mobile phase A was 0.1% formic acid in water, and mobile phase B was 0.1% formic acid in acetonitrile. Following equilibration of the chromatographic column with 100% mobile phase A, the sample was directly loaded via an autosampler and subjected to gradient elution over a 6.9 min duration. Mass spectrometric analysis was performed using data-independent acquisition (DIA-PASEF) mode in positive ion detection, with a total analysis time of 7 min. The primary full-scan range was set at 380–980 *m*/*z* with a resolution of 240,000, employing an isolation window of 2 *m*/*z* and a normalized collision energy (NCE) of 25%. The secondary full-scan range covered 150–2000 *m*/*z*.

#### 2.4.5. Protein Identification

Raw MS files were processed using Spectronaut software (v19.0.240604.62635) with the Pulsar search engine. Data were searched against the UniProt Bos taurus FASTA database (uniprotkb_iRT_Bos_taurus_9913_2025_03_05.fasta). Carbamidomethylation (C) was set as a fixed modification, and methionine oxidation and protein N-terminal acetylation were set as variable modifications. Trypsin was specified as the digestion enzyme, allowing up to two missed cleavages. The false discovery rate (FDR) for peptide-spectrum matches (PSMs) and peptides was controlled at 1%. The initial precursor and fragment mass tolerances were both set at 20 ppm. All other parameters were kept at default settings.

### 2.5. Proteomics Data Analysis

Data preprocessing involved the following steps: (1) filtering by unique peptide count, retaining proteins with ≥1 unique peptide; (2) imputation of missing values using half of the minimum observed value; (3) after preprocessing, 1690 proteins were retained. Use R (ggplot2, V3.3.5) to draw a Venn diagram with the names of the proteins identified in each group. Use R (ggplot2, V3.3.5) to sort the proteins identified in all groups according to their abundance and draw a scatter plot for the distribution analysis of protein abundance. Data were log-transformed and mean-centered using R (version 3.6.3) or SIMCA (v18.0.1, Sartorius Stedim Data Analytics AB, Sweden), followed by PCA modeling. PCA score plots were generated using R (ropls, ggplot2). Differentially expressed proteins (DEPs) were identified based on chi-square test *p* < 0.05 and fold change ≤ 0.5 or ≥ 2. Volcano plots were generated using R (ggplot2). Proteins with *p* < 0.01 and fold change ≤ 0.1 or ≥ 100 were further selected for hierarchical clustering (heatmaps with R pheatmap), radar plot visualization (R fmsb), and COG (KOG) functional classification (bar charts with R ggplot2). GO enrichment analysis was performed by mapping genes to the Bos taurus Gene Ontology database (http://www.geneontology.org/ (accessed on 17 May 2026)) and visualized using R (topGO, ggplot2). KEGG pathway analysis was conducted using the KEGG REST API (https://rest.kegg.jp (accessed on 17 May 2026)), with visualization in R (ggplot2).

### 2.6. Integrated Multi-Omics Analysis

The information of 24 metabolites with significant differences in lipidomics and 17 proteins with significant differences in proteomics was statistically analyzed. Spearman correlation analysis was conducted to assess the associations between differential metabolites and proteins, using R (version 4.3.3), and the correlation heatmap was plotted with pheatmap 1.0.12. The up and downregulation profiles of differential metabolites and proteins were integrated, and all pathways mapped to Bos taurus were retrieved from the Kyoto Encyclopedia of Genes and Genomes (KEGG) database. Pathways jointly enriched by both proteomics and metabolomics datasets were identified, and the results of KEGG enrichment were visualized as bar plots using R (version 3.3.2). O2PLS analysis and the associated nine-quadrant plot were performed in R (version 4.5.2). O2PLS modeling was conducted using the OmicsPLS package (version 2.1.0), and graphical visualization was performed using ggplot2 (version 4.0.3). For the nine-quadrant association analysis, Pearson correlation coefficients were calculated to quantify the linear associations between the two omics datasets, and the results were visualized using ggplot2.

### 2.7. α-Ketoglutarate Quantification

For the measurement of α-ketoglutarate, 100 μL of bovine milk sample was mixed with 1 mL of methanol, vortexed for 3 min, and sonicated for 30 min. Samples were centrifuged at 3000× *g* for 30 min, and the supernatant was filtered through a 0.45 μm membrane filter. Standard solutions of α-ketoglutarate were prepared in parallel. The standard solution and test samples were analyzed using a liquid chromatography–tandem mass spectrometry (LC-MS/MS) system (Agilent 1290-G6470, Santa Clara, CA, USA). Quantitative calculations were performed based on the measured chromatographic peak-area response values. Analytical conditions were as follows: a C18 column was employed, and the mobile phase consisted of solvent A (methanol) and solvent B (0.05% (*v*/*v*) ammonium formate). The mobile phase was delivered at a flow rate of 0.3 mL/min, with the column temperature set at 30 °C and an injection volume of 20 μL. α-Ketoglutarate (AKG) was identified using multiple reaction monitoring (MRM) in positive ion mode. The desolvation gas temperature and flow rate were maintained at 350 °C and 10 L/min, respectively. The nebulizer pressure was set to 30 psi. The capillary voltage was configured at 4000 V, and the collision cell accelerator voltage was 3 V. MRM parameters included a precursor ion peak at *m*/*z* 145.00, with fragment ion peaks at *m*/*z* 100.90 and *m*/*z* 57.00. Spearman correlation analysis was conducted between α-ketoglutarate and metabolites and proteins using R (version 4.3.3), and the correlation heatmap was plotted using pheatmap 1.0.12.

## 3. Results

### 3.1. The Outcomes of Metabolite Analysis of Colostrum and Mature Milk from Primiparous Holstein Cows

**Figure 1:**
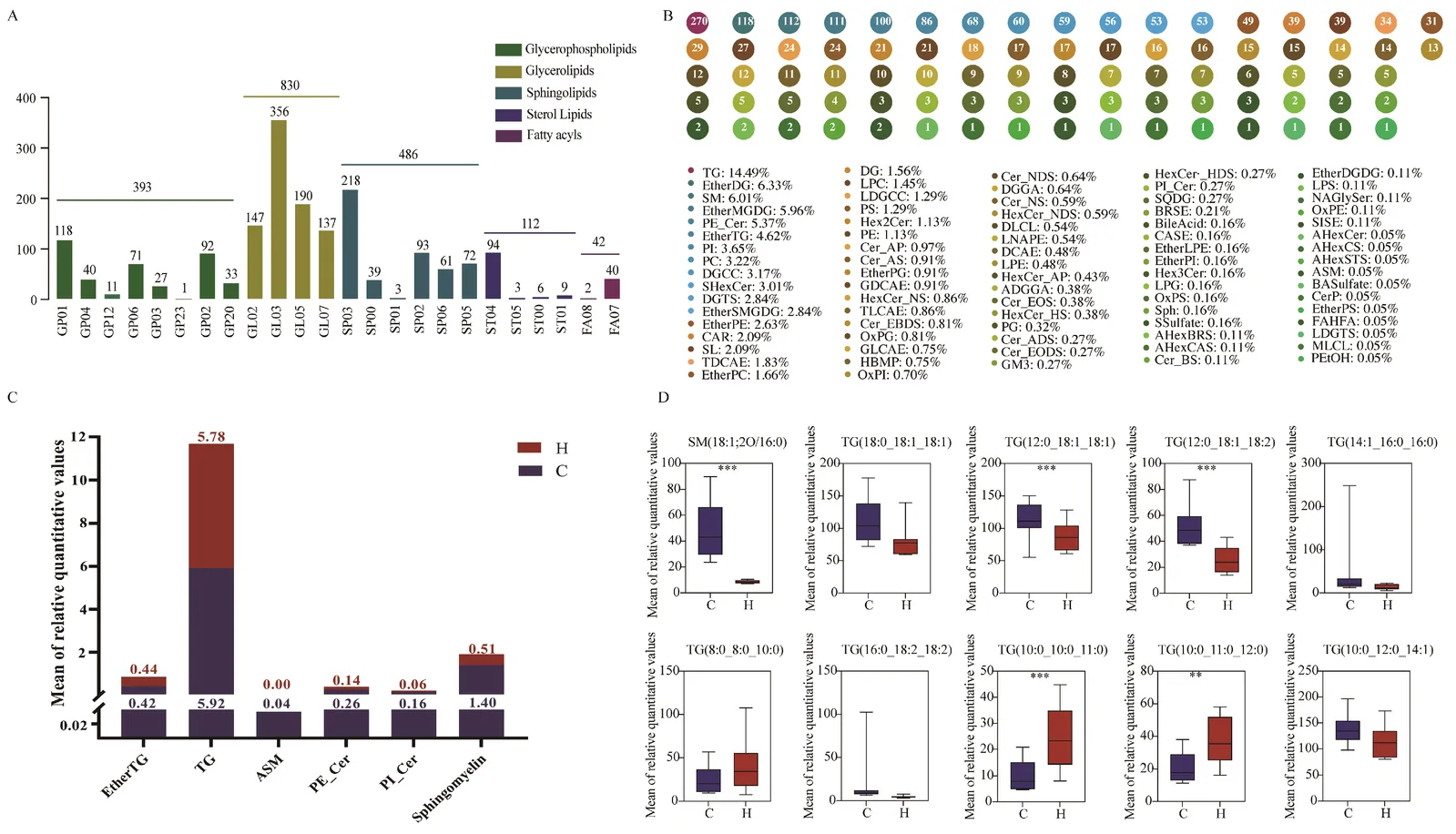
Analysis of metabolite components in colostrum (C group) and mature milk (H group) from primiparous Holstein cows. (**A**) Number of identified metabolites across five major lipid categories: glycerophospholipids (GP), glycerolipids (GL), sphingolipids (SP), sterol esters (ST), and fatty acyls (FA). (**B**) Relative proportion of 82 lipid subclasses in the total lipidome. (**C**) Relative abundance of triacylglycerols (GL03) and phosphosphingolipids (SP03) between colostrum and mature milk. (**D**) Quantitative comparison of the top 10 differential lipid species within TG and SM subclasses between colostrum and mature milk. Data are presented as mean ± SD. ** *p* < 0.01, *** *p *< 0.001, *p* < 0.05 was considered statistically significant.

A total of 1863 lipid metabolites were identified in colostrum and mature milk from eight primiparous Holstein cows. They were primarily classified into five major categories: glycerophospholipids [GP] (393), glycerides [GL] (830), sphingolipids [SP] (486), sterol lipids [ST] (112), and fatty acyls [FA] (42). As shown in Figure 1A, triacylglycerols [GL03] and phosphosphingolipids [SP03] have the largest abundance among the metabolites in Holstein cow milk. As to the metabolite distribution, TG accounts for the highest proportion at 14.5%, followed by Ether DG at 6.3% and SM at 6.0% (Figure 1B). Additionally, by summing the quantitative intensities of all lipid species within each subclass, we calculated their relative abundance between lactation stages. As shown in Figure 1C, TG, SM, and phosphatidylinositol-ceramide (PI-Cer) exhibited significant differences between colostrum and mature milk with relative abundance ratios of 0.14, 0.89, and 0.12, respectively. Furthermore, the top 10 metabolites showing the greatest differential expression within the TG and SM classes were selected for comparative analysis based on their relative quantification. As shown in Figure 1D, SM (18:1;2O/16:0), TG (12:0_18:1_18:1) and TG (12:0_18:1_18:2) exhibited significantly higher relative expression in colostrum than in mature milk (*p* < 0.05), whereas TG (10:0_10:0_11:0) and TG (10:0_11:0_12:0) showed significantly lower relative expression in colostrum compared to mature milk (*p* < 0.05).

### 3.2. Multivariate Statistical Analysis of Lipid Metabolites in Colostrum and Mature Milk of Primiparous Holstein Cows

**Figure 2:**
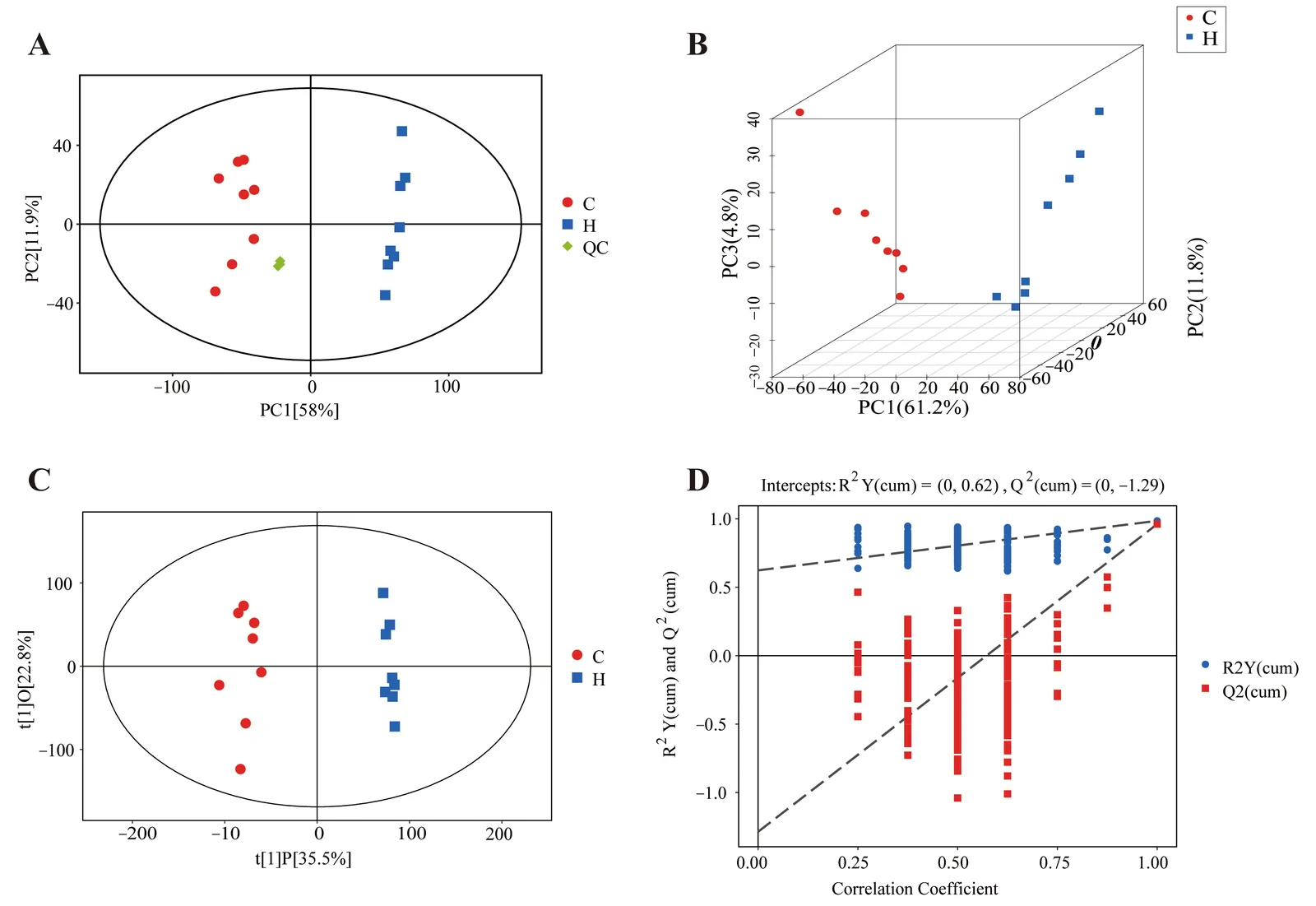
Multivariate statistical analysis of lipidomics. (**A**) PCA score plot of all milk samples, including all analyzed samples and QC samples. (**B**) Three-dimensional PCA score plot comparing colostrum (C group) and mature milk (H group). (**C**) OPLS-DA score plot between colostrum and mature milk. t[1]P represents the predicted principal component score of the first principal component, which reveals the differences between sample groups. t[1]O represents the orthogonal principal component score, which shows the differences within sample groups. (**D**) Permutation test plot (200 permutations) for OPLS-DA model validation.

To further investigate the differences in lipid metabolites between colostrum and mature milk, multivariate statistical analyses were performed, including unsupervised principal component analysis (PCA) and supervised orthogonal partial least squares–discriminant analysis (OPLS-DA). As shown in Figure 2A, all samples were located within the 95% confidence ellipse of the PCA model, indicating a high similarity in most lipid species between colostrum and mature milk. Nevertheless, samples were still separated along PC1 (58%), suggesting the presence of substantial differences in lipid composition. PC2 = 11.9%, the samples show varying degrees of separation along the PC2 axis, indicating the representativeness of the selected samples and the stability of the detection process. As shown in Figure 2B (PC1 = 61.2%, PC2 = 11.8%, PC3 = 4.8%), these results were further confirmed. As shown in Figure 2C, the OPLS-DA model revealed a predictive component score of t1p (35.5%) and an orthogonal component score of t1o (22.8%), further confirming compositional differences in lipid metabolites between colostrum and mature milk, which was consistent with the PCA results. Additionally, a corresponding OPLS-DA model was constructed using 200 permutation tests. The R^2^ and Q^2^ values of the random models were 0.62 and −1.29, respectively, indicating model reliability and the absence of overfitting (Figure 2D).

### 3.3. Identification and Screening of Biomarkers Among Differential Metabolites Between Colostrum and Mature Milk

**Figure 3:**
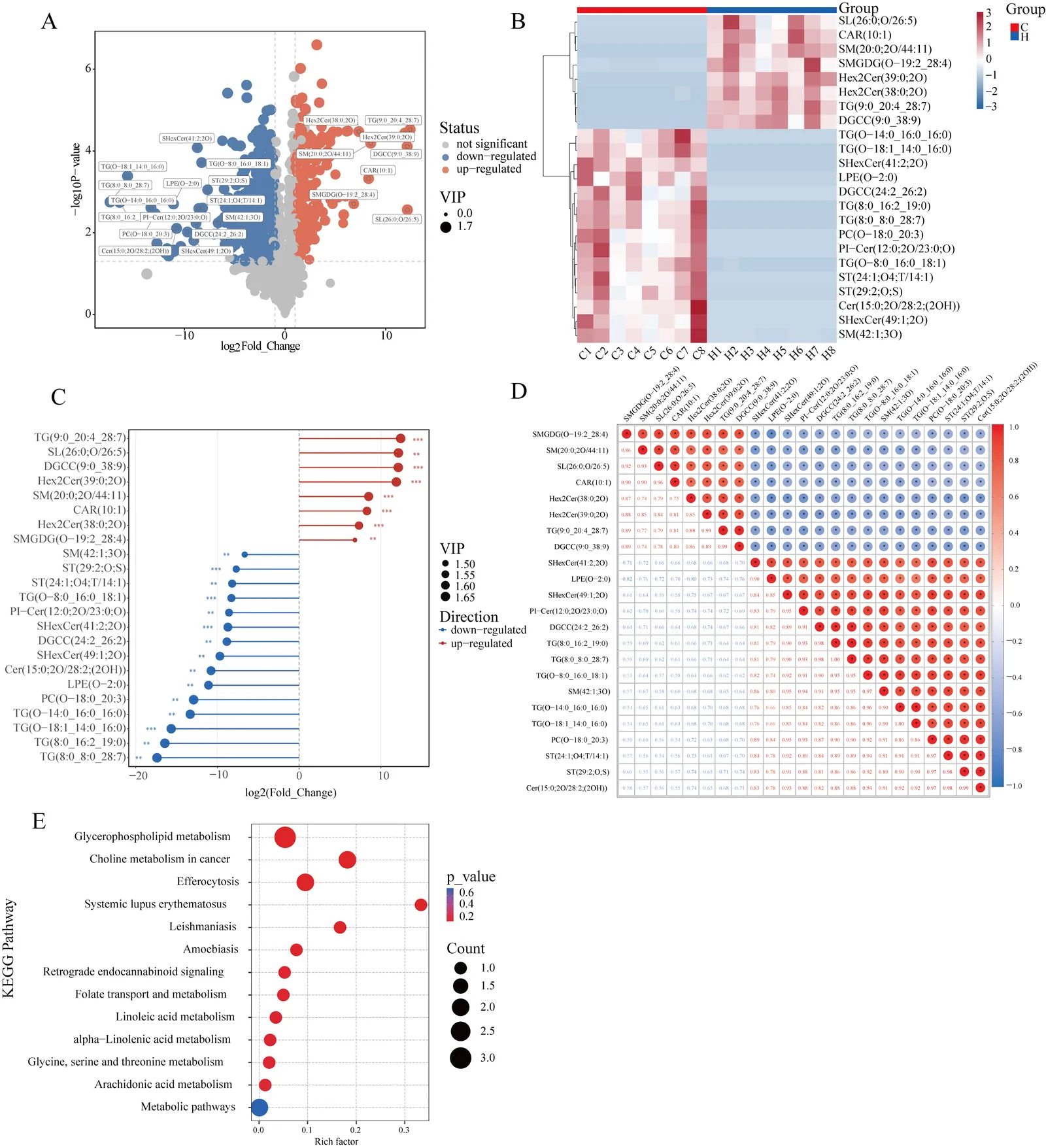
Identification and screening of differential metabolites. (**A**) Volcano plot of differential metabolites. (**B**) Hierarchical clustering heatmap comparing colostrum (C group) and mature milk (H group). (**C**) Lollipop plot of top differential metabolites. (**D**) Correlation heatmap of differential metabolites. (**E**) KEGG pathway enrichment bar plot. * *p* < 0.05, ** *p* < 0.01, *** *p *< 0.001, *p* < 0.05 was considered statistically significant.

To identify potential biomarkers that distinguish colostrum from mature milk, univariate statistical analyses (Paired *t*-test and fold-change analysis) were performed in combination with multivariate statistical results. Differential metabolites were screened based on the following criteria: variable importance in projection (VIP) score from the first principal component of the OPLS-DA model >1.0, *p* < 0.05, and fold change (FC < 0.5 or FC > 2). A total of 945 differential lipid metabolites were identified across the five major lipid classes. As shown in Figure 3A, in colostrum, 272 metabolites were significantly upregulated (*p* < 0.05, VIP > 1, FC > 2) and 673 were significantly downregulated (*p* < 0.05, VIP > 1, FC < 0.5) compared with mature milk. Upon further screening of differential metabolites, we identified eight substantially elevated metabolites (*p* < 0.01, VIP > 1, FC > 100) and 15 downregulated metabolites (*p* < 0.01, VIP > 1, FC < 0.01) (Figure 3B) in colostrum compared to the mature milk. As shown in Figure 3C, TG (9:0_20:4_28:7) exhibited the largest fold change among upregulated metabolites, whereas TG (8:0_8:0_28:7) exhibited the largest decrease, both belonging to glycosyl triacylglycerols [GL03]. Correlation analysis showed strong correlations among upregulated and downregulated metabolites, with DGCC (9:0_38:9) strongly correlated with TG (9:0_20:4_28:7) (r = 0.99) and Cer (15:0;2O/28:2; (2OH)) strongly correlated with ST (29:2; O; S) (r = 0.99) (Figure 3D). The correlation coefficient between the upregulated metabolite DGCC (9:0_38:9) and the downregulated metabolite LPE (O-2:0) is the largest, which is −0.80. KEGG enrichment analysis was performed on all the differential metabolites, and a total of 13 pathways were screened out. Among them, the Glycerophospholipid metabolism pathway was enriched with the largest number of differential metabolites, which was three (Figure 3E).

### 3.4. The Outcomes of Proteomic Analysis of Colostrum and Mature Milk in Primiparous Holstein Cows

**Figure 4:**
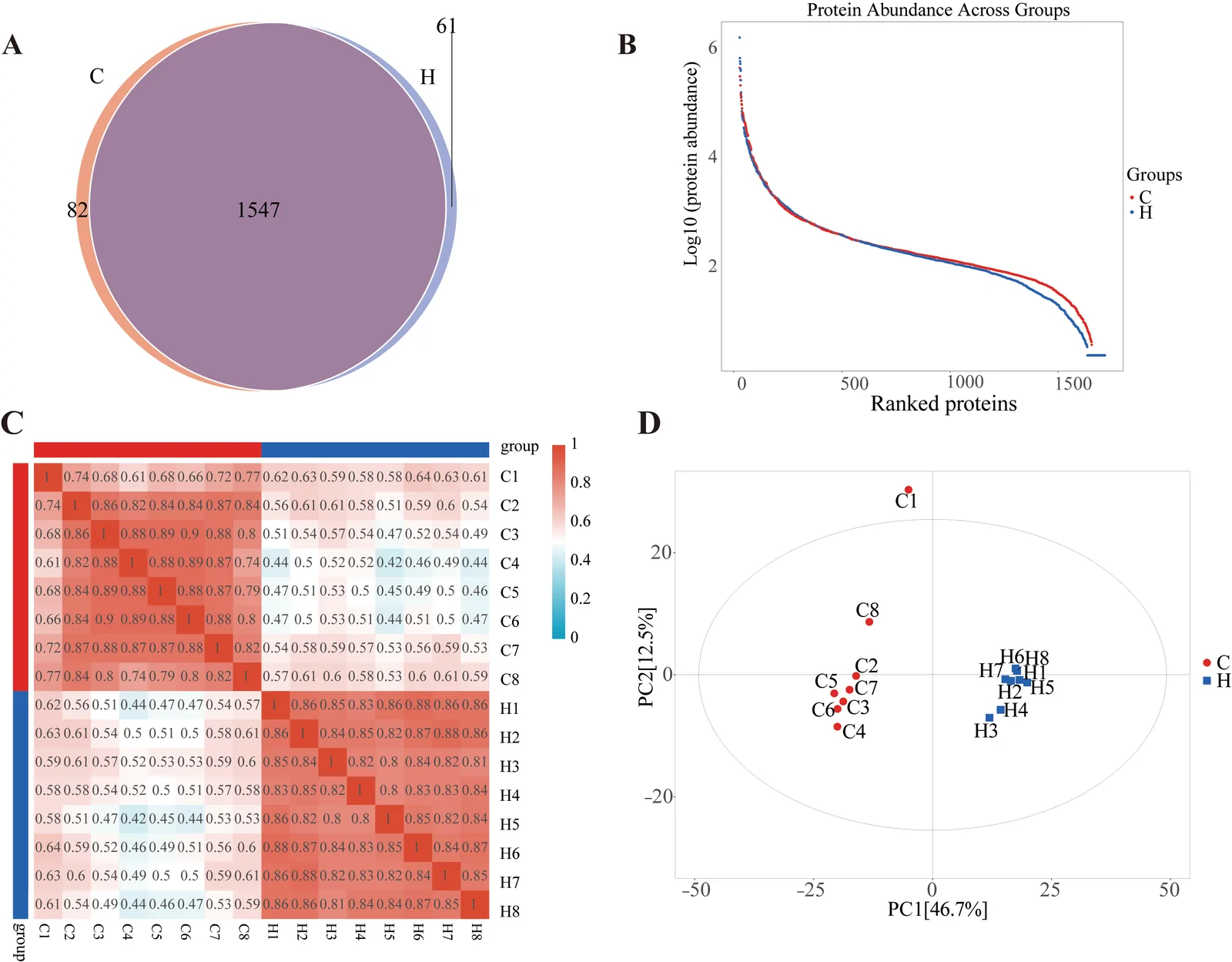
The general distribution of proteins between colostrum and mature milk analyzed from proteomics data. (**A**) Venn diagram of protein overlaps between groups. (**B**) Scatter plot of protein abundance distribution. (**C**) Heatmap of sample-to-sample correlation. (**D**) PCA score plot of all samples. Note: C group = colostrum; H group = mature milk.

Proteomic profiling was performed to investigate the differences in protein composition between colostrum and mature milk in primiparous Holstein cows. A total of 1690 proteins were identified, of which 1547 were common between colostrum and mature milk, while 82 were uniquely expressed in colostrum, and 61 were uniquely expressed in mature milk (Figure 4A). Protein ranking by abundance revealed that, within the abundance range of 1000–1500, colostrum exhibited higher protein abundance than mature milk (Figure 4B). Pearson correlation analysis showed that the correlation coefficients between colostrum and mature milk samples ranged from 0.44 to 0.64, with intra-colostrum sample correlations exceeding 0.61 and intra-mature milk sample correlations exceeding 0.80 (Figure 4C). Principal component analysis (PCA) was conducted to visualize the overall variance in protein profiles. PC1 and PC2 explained 46.7% and 12.5% of the total variance, respectively. All 16 experimental samples were included in the PCA and 15 samples fell within the 95% confidence ellipse, whereas one colostrum sample (C1) was located outside the 95% confidence ellipse. Importantly, this sample was retained in all subsequent analyses (Figure 4D). * *p* < 0.05, ** *p* < 0.01, *** *p *< 0.001.

### 3.5. The Differentially Expressed Proteins Between Colostrum and Mature Milk in Primiparous Holstein Cows

**Figure 5:**
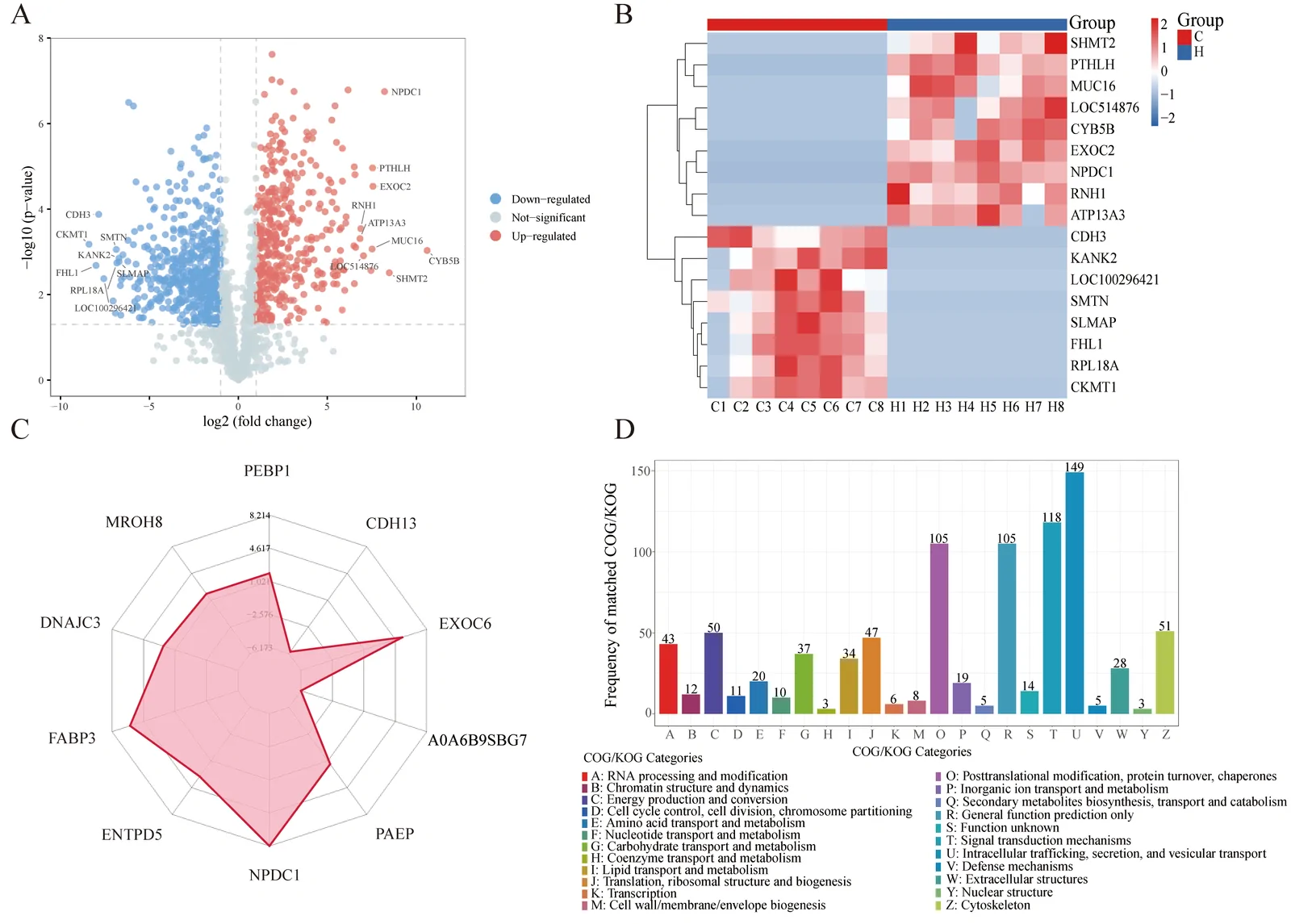
Analysis of differentially expressed proteins (DEPs). (**A**) Volcano plot of DEPs between colostrum (C group) and mature milk (H group). (**B**) Hierarchical clustering heatmap of DEPs. (**C**) Radar chart of fold-change distribution of DEPs. (**D**) Histogram of Clusters of Orthologous Groups (COG) functional classification.

The differentially expressed proteins (DEPs) were screened based on the criteria of *p* < 0.05 and FC < 0.5 or >2. A total of 933 DEPs were identified, including 408 up- and 525 downregulated proteins. To identify the potential biomarker proteins between colostrum and mature milk, a more stringent threshold (*p* < 0.01 and FC > 100 or FC < 0.01) was applied, resulting in nine markedly up- and eight markedly downregulated proteins, as indicated in Figure 5A. Hierarchical clustering analysis revealed clear segregation of the two groups, with the nine upregulated proteins predominantly expressed in mature milk and the eight downregulated proteins mainly enriched in colostrum (Figure 5B). The fold-changes in upregulated proteins ranged from 5.85 to 10.611, whereas those of downregulated proteins ranged from −3.652 to −8.406 (Figure 5C). Functional classification of DEPs using the Clusters of Orthologous Groups (COG) database indicated that most DEPs were enriched in signal transduction mechanisms (148 proteins) and intracellular trafficking, secretion, and vesicular transport pathways (158 proteins). In contrast, three proteins were enriched in the Coenzyme transport and metabolism pathway and three proteins were enriched in the nuclear structure pathway, respectively (Figure 5D).

### 3.6. The Distribution of Differentially Expressed Proteins with GO and KEGG Enrichment Analysis Between Colostrum and Mature Milk

**Figure 6:**
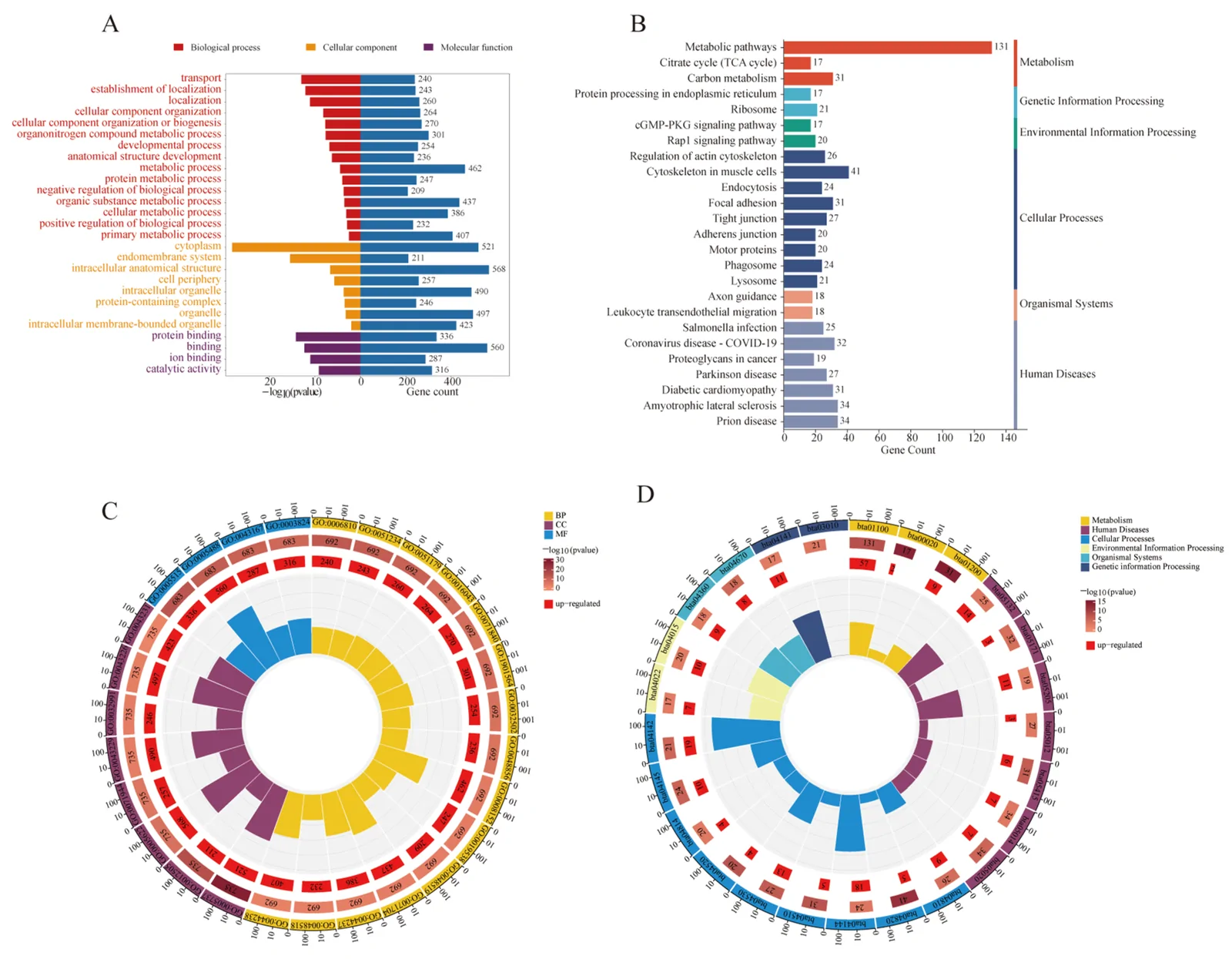
GO and KEGG enrichment analysis of differentially expressed proteins. (**A**) GO enrichment classification bar plot of differentially expressed proteins; (**B**) KEGG pathway enrichment bubble plot of differentially expressed proteins; (**C**) GO enrichment chord plot of differentially expressed proteins; (**D**) KEGG pathway enrichment chord plot of differentially expressed proteins.

GO enrichment analysis was performed on the 933 differentially expressed proteins (DEPs), yielding 1379 terms in the Biological Process (BP) category, 278 terms in the Molecular Function (MF), and 252 terms in the Cellular Component (CC). Based on the criteria of *p* < 0.01 and Count > 200, 15 BP terms, eight CC terms, and four MF terms were retained (Figure 6A). In the BP group, the GO:0008152 metabolic process was enriched to the largest number of proteins, which were 462; among them, 199 proteins were upregulated. In the CC group, the GO:0005622 intracellular anatomical structure was enriched to the largest number of proteins, which was 568; among them, 253 proteins were upregulated. In the MF group, GO:0005488 binding was enriched to the largest number of proteins, which were 560; among them, 244 proteins were upregulated, as shown in Figure 6C. KEGG pathway enrichment analysis was performed on the differentially expressed proteins, and a total of 317 pathways were identified. Based on the screening criteria (*p* < 0.01 and Count > 17), 27 significant pathways were obtained, which were significantly enriched in the Cellular Processes category, as shown in Figure 6B. Among them, the bta00010 pathway had the largest number of enriched genes, which were 131, with 57 upregulated genes. The bta03010 pathway had 21 enriched genes, all of which were downregulated, as shown in Figure 6D.

### 3.7. The Potential Correlations Between Proteomics and Metabolomics with Integrative Analysis

**Figure 7:**
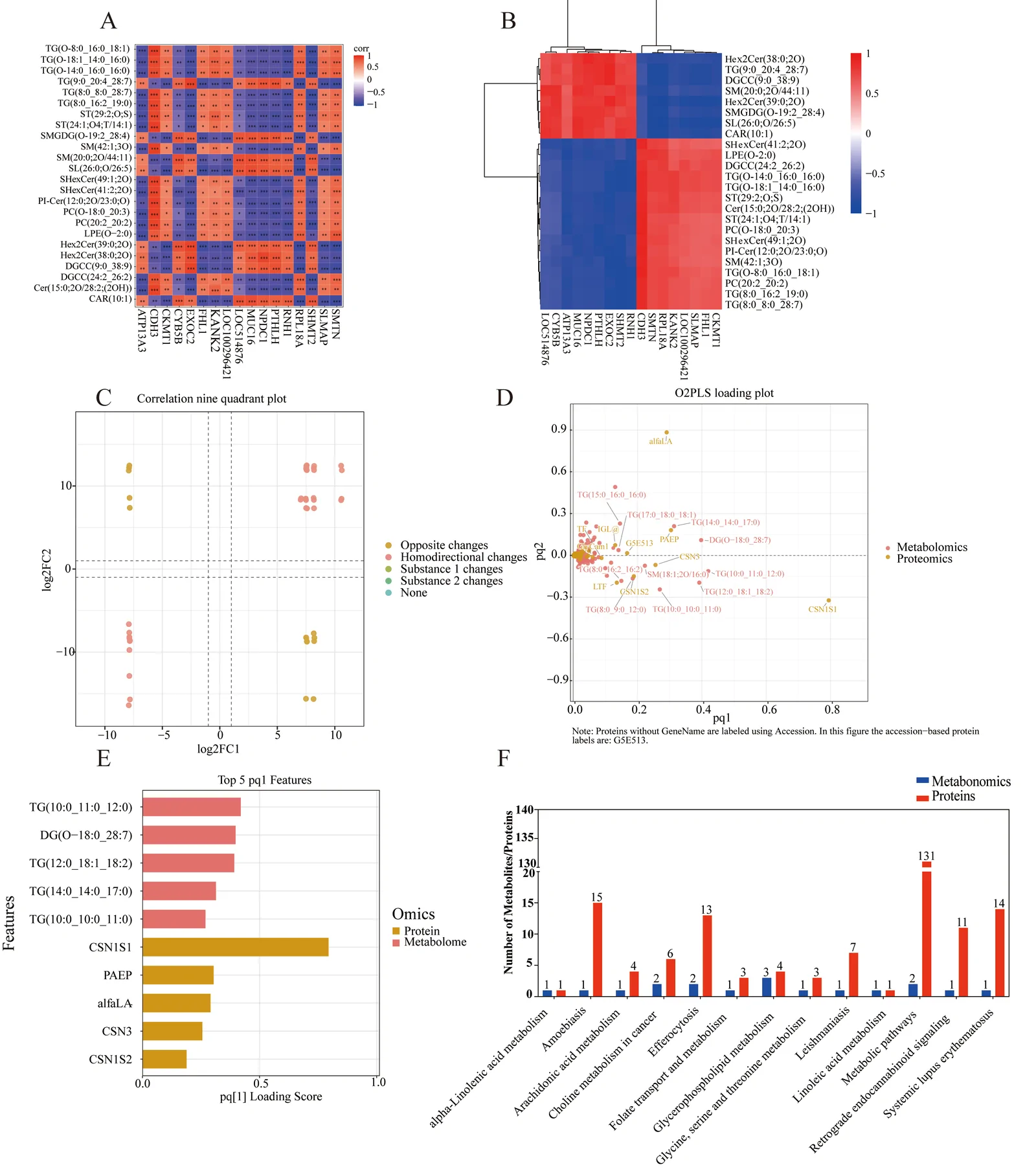
Integrated proteomic and metabolomic analysis. (**A**) Correlation heatmap of colostrum (C group) and mature milk (H group); (**B**) correlation clustering heatmap of colostrum (C group) and mature milk (H group); (**C**) nine-quadrant plot of associations between differentially expressed proteins and metabolites. Note: the *X*-axis represents the log2 fold change in differentially expressed proteins, and the *Y*-axis represents the log2 fold change in differentially expressed metabolites. Points located in quadrants 1 and 9 (Opposite changes) indicate opposite trends of change between proteins and metabolites; quadrants 3 and 7 (Homodirectional changes) indicate consistent trends; quadrants 2 and 8 (Substance 1 changes) indicate changes only in metabolites; quadrants 4 and 6 (Substance 2 changes) indicate changes only in proteins; and quadrant 5 (None) represents no significant changes in either component. (**D**) O2PLS loading plot. Note: the *X*-axis represents the explanation of the first principal component, and the *Y*-axis represents the explanation of the second principal component. Different colors represent different omics datasets. The plot shows the relationships between variables and principal components, where the absolute value of the loading reflects the strength of association between proteomic and metabolomic datasets. (**E**) O2PLS feature importance ranking plot. Note: the pq[1] Loading Score indicates the magnitude of the first principal component value. The first color represents lipidomics, and the second color represents proteomics. Higher bars indicate greater importance, and the top 20 features are shown. (**F**) Bar plot of KEGG pathways jointly enriched by differentially expressed proteins and metabolites in colostrum and mature milk. * *p* < 0.05, ** *p* < 0.01, *** *p *< 0.001.

To test whether there is a potential correlation between proteomics and metabolomics, 23 significantly differential metabolites and 17 significantly differential proteins were selected for integrative analysis. The results showed that all 23 differential metabolites were significantly correlated with the 17 differential proteins (Figure 7A). Among them, eight upregulated metabolites were significantly correlated with nine upregulated proteins, while 15 downregulated metabolites were significantly correlated with eight downregulated proteins (Figure 7B). Pearson correlation analysis was conducted between the differential metabolites and proteins. Among the 23 differential metabolites and 17 differential proteins, there were 33 positive correlations and 14 negative correlations. Among them, the CDH3 protein had the most differential metabolite-related pairs, including 10 positive correlations and five negative correlations. SM (20:0;2O/44:11) was correlated with six proteins, including five positive correlations and one negative correlation (Figure 7C). Two-way orthogonal partial least squares (O2PLS) analysis was conducted on 945 differential metabolites and 933 differential proteins. There were 10 metabolites and four proteins with the pq1 greater than 0.2, and five metabolites and one protein with the pq2 greater than 0.2 (Figure 7D). Based on the first principal component values, TG (10:0_11:0_12:0) and P02662 (CSN1S1) were identified as the most important features in the metabolome and proteome, respectively (Figure 7E). Based on the Kyoto Encyclopedia of Genes and Genomes (KEGG) pathway database, a combined analysis of lipidomics and proteomics data was conducted, and a total of 13 significantly enriched pathways were screened out. Among them, the Metabolic pathways enriched the largest number of proteins, reaching 131 types, and simultaneously enriched two kinds of metabolites; the Glycerophospholipid metabolism pathway enriched three kinds of metabolites and four kinds of proteins (Figure 7F). These two pathways are very likely to represent the biological pathways with the most significant metabolic differences between colostrum and mature milk.

### 3.8. The α-Ketoglutarate Level and Its Relation with Differentially Expressed Proteins and Metabolites in Colostrum and Mature Milk

**Figure 8:**
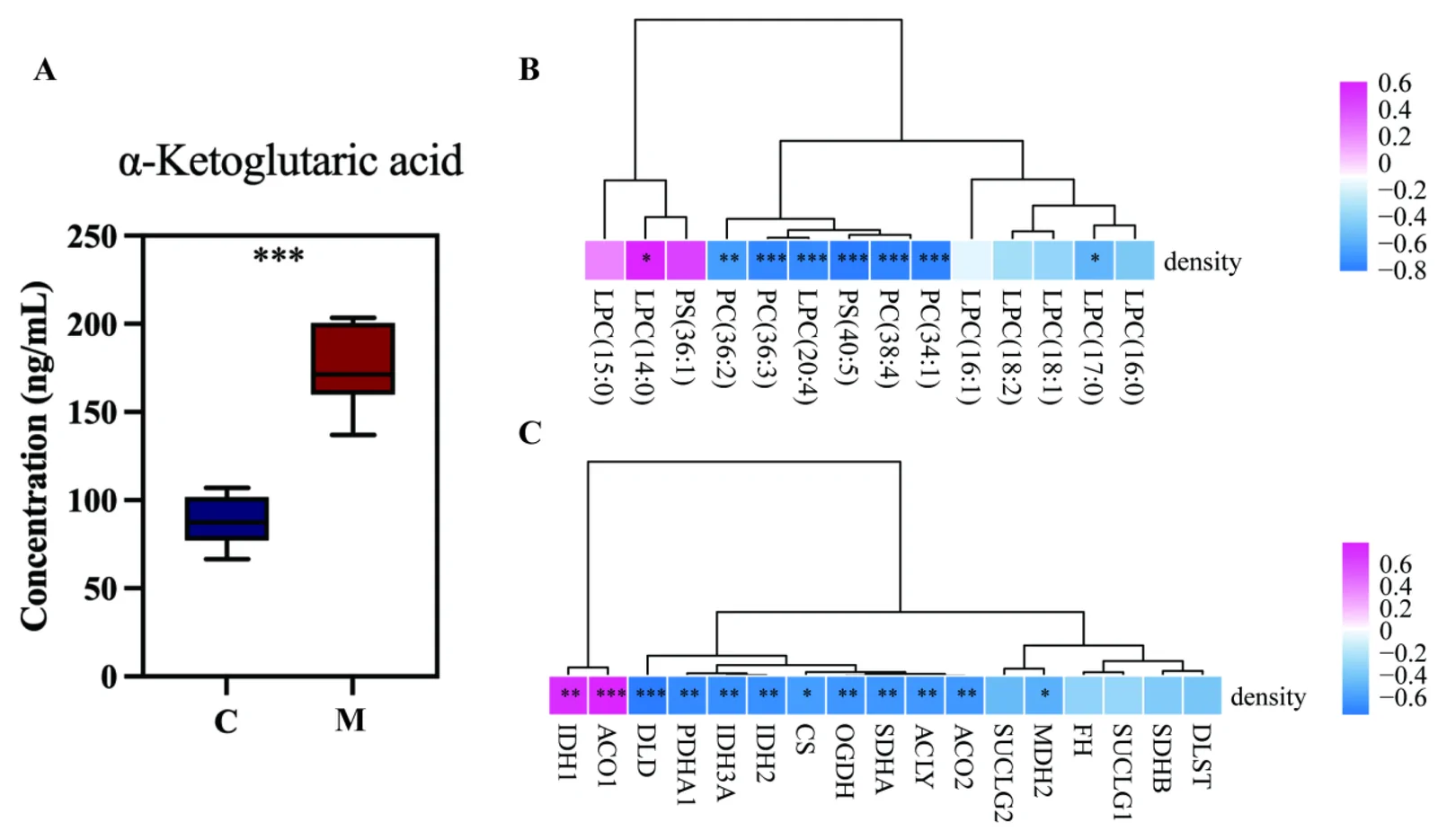
Correlation analysis of α-ketoglutarate with differentially expressed proteins and metabolites. (**A**) Concentration of α-ketoglutarate in milk; (**B**) correlation analysis between α-ketoglutarate and differentially expressed metabolites; (**C**) correlation analysis between α-ketoglutarate and differentially expressed proteins involved in the TCA cycle pathway. * *p* < 0.05, ** *p* < 0.01, *** *p *< 0.001.

Finally, we measured the content of α-ketoglutaric acid in colostrum and mature milk. The results showed that the content of ketoglutaric acid in colostrum was 88.50 ng/mL, which was two folds lower than that in mature milk (175.89 ng/mL) (Figure 8A). Based on the data of Figure 3E, the 13 KEGG pathways were enriched in the differential metabolites, mainly involving C0423, C00157, and C02737 in the KEGG COMPOUND database. Therefore, the correlation analysis was conducted between the 14 differential metabolites in these three pathways with α-ketoglutarate. The results showed that α-ketoglutarate was significantly correlated with LPC (14:0), PC (36:2), PC (36:3), LPC (20:4), PS (40:5), PC (38:4), and LPC (17:0) (Figure 8B). In the differential protein pathways, the Citrate cycle (TCA cycle) pathway related to α-ketoglutarate metabolism was enriched (Figure 6B). The correlation analysis between the differential proteins in the pathway and α-ketoglutarate was conducted. The result showed that α-ketoglutarate was significantly correlated with IDH1, ACO1, DLD, PDHA1, IDH3A, IDH2, CS, OGDH, SDHA, ACLY, ACO2, and MDH2 (Figure 8C).

## 4. Discussion

It is well known that colostrum is rich in bioactive components, including maternal antibodies, which can improve the immune activity of calves. These bioactive components will be altered during different lactation stages, with the mature milk having different quality compared to the colostrum. Many studies have compared the components between colostrum and mature milk by traditional methods and obtained variable results due to the methodological limitations [19]. In this study, for the first time, lipidomics and data-independent acquisition (DIA) quantitative proteomics were used to systematically analyze the metabolite and protein compositions of colostrum and mature milk in primiparous Holstein cows. These analyses can identify majority of the metabolites and proteins and Thyer differential patterns in colostrum and mature milk. The results allow us to screen out the most differential factors with jointing analysis to clarify their potential association and biomarkers to distinguish them. The results showed that the lipidomics analysis had identified a total of 1863 lipid metabolites, mainly including five major categories: glycerophospholipids, glycerides, sphingolipids, sterol esters, and fatty acyls. Among them, the triacylglycerols (TG, GL03) and phosphosphingolipids (SP03) were the most abundant metabolites. Triacylglycerols, formed by esterification of glycerol with three fatty acids, play a central role in energy storage, metabolic regulation, and physiological functions [20]. Phosphosphingolipids, as the major subclass of sphingolipids, are essential structural components of cell membranes and participate in the regulation of multiple cellular-signal-transduction processes [21]. Based on the previous reports on bovine milk compositions [22,23,24], we identified 10 molecules with the most significant differential expression among TG, sphingomyelin (SM), and phosphoinositol ceramide (PI_Cer). In the comparative analysis of colostrum and mature milk, four lipid molecules showed significant differentiation, i.e., SM (18:1;2O/16:0), triglyceride TG (12:0_18:1_18:1), and TG (12:0_18:1_18:2) were significantly higher and TG (10:0_10:0_11:0) and TG (10:0_11:0_12:0) were significantly lower in colostrum compared to mature milk (*p* < 0.05).

To further analyze the differences in lipid metabolites between colostrum and mature milk of primiparous dairy cows, we conducted multivariate and univariate statistical analyses on these identified differential metabolites. The results of principal component analysis (PCA) and orthogonal partial least squares discriminant analysis (OPLS-DA) indicate that although most of the lipid species in colostrum and mature milk are highly similar, several lipid metabolites were highly differentiated between colostrum and mature milk. Previous studies have reported a total of 851 lipid molecules by using quantitative lipidomics analysis on yak colostrum and mature milk, in which 43 were significantly differentiated based on the criteria of (VIP > 1, FC < 0.5 or >2 and *p* < 0.05). In this study, a total of 945 differential metabolites were screened out. To identify potential biomarkers, a more stringent screening standard (VIP > 1, FC ≤ 0.01 or FC ≥ 100 and *p* < 0.01) was used, and ultimately, eight significantly upregulated and 15 significantly downregulated lipid metabolites were identified in colostrum compared to mature milk. Out of the 23 differentially expressed metabolites, nine were glycerolipids. Among them, the upregulated TG (9:0_20:4_28:7) and the downregulated TG (O-8:0_16:0_18:1) showed the most significant differences, and both were classified as glycosyl triacylglycerols [GL03]. These 23 differentially expressed lipid metabolites were enriched in 13 pathways, with three metabolites being enriched in Glycerophospholipid metabolism. The downstream metabolites of glycerophospholipids such as IP_3_, DAG, PA, and AA act as important second messengers, regulating intracellular calcium mobilization, PKC activation, cell proliferation, apoptosis, and other signal cascade processes [25,26]. Colostrum contains a relatively large amount of glycerophospholipids, indicating its importance in promoting the development of the central nerve system of young animals.

Milk is also rich in proteins and peptides. Proteomics technology has been widely applied in the analysis of milk and other dairy products [27]. By using this technology, 283 and 159 proteins in the whey and 593 and 349 proteins in the milk fat globule membrane (MFGM) fraction were identified in Guanzhong dairy goats and Holstein dairy cows, respectively [16]. In this study, a total of 1690 proteins were identified using quantitative proteomics technology. Among them, 82 proteins were specifically expressed in colostrum and 61 proteins were specifically expressed in mature milk. Quantitative analysis showed that the protein abundance in colostrum was significantly higher than that in mature milk. Previously, a study utilized label-free proteomics technology to identify XDH as a potential biomarker for milk. In this study, a total of 933 differentially expressed proteins were screened out based on the conditions of *p* < 0.05 and FC < 0.5 or FC > 2. To more accurately screen out the potential signature proteins, we applied more stringent screening criteria: (*p* < 0.01, FC > 100 or FC < 0.01). Finally, 17 significantly differentiated proteins were identified, with nine significantly upregulated in mature milk, while eight significantly downregulated in colostrum. COG functional annotation was conducted for 933 differentially expressed proteins. Among them, 118 were significantly enriched in signal transduction mechanisms, suggesting that these proteins are involved in endocrine, neuroimmune regulation and other processes, while 149 were enriched in the intracellular trafficking, secretion, and vesicular transport pathway, which mainly involves the loading, transportation, secretion of intracellular substances and the transfer across organelles [28]. All of these pathways are closely related to the development of the nervous system and immune function in newborn calves.

The quality and quantity of proteins determine the nutritional value of milk. In this study, we conducted GO and KEGG enrichment analyses, respectively, on the 933 differentially expressed proteins. In the GO enrichment analysis, a total of 1379 pathways were identified in the Biological Process group. Among them, 462 proteins were significantly enriched in metabolic-related processes (GO:0008152), indicating these differentially expressed proteins participating in regulating metabolic activities [29]. A total of 278 pathways were screened in the Molecular Function group, and a total of 560 proteins were significantly enriched in binding (GO:0005488), reflecting that the differentially expressed proteins have a strong binding function, which is the basis of various biological processes [30]. The Cellular Component (CC) group identified 252 pathways, among which 568 proteins were mainly concentrated in intracellular anatomical structures (GO:0005622), indicating that the differential proteins play a significant role in maintaining the structure and function of organelles [31]. Meanwhile, KEGG pathway analysis identified a total of 317 enriched pathways. Based on the screening criteria of *p* < 0.01 and Count > 17, a total of 25 significant pathways were identified. The differentially expressed proteins were mainly enriched in cell process-related pathways, indicating that intracellular transport, metabolic regulation and signal transduction are the core areas of functional changes, which is consistent with the results of Gene Ontology (GO) enrichment analysis. This CO analysis uncovered 131 proteins which were enriched in Metabolic pathways (bta01100), suggesting a strong link between protein function and energy metabolism [32]. All 21 genes in the ribosome pathway (bta03010) showed a trend of downregulation, and this might be one of the key regulatory factors leading to the differences in some proteins and metabolites between colostrum and mature milk [33].

In recent years, omics technologies such as genomics, transcriptomics, and proteomics have been increasingly applied in dairy science. For instance, combined proteomic and lipidomic analyses have revealed the comparative characterization and functional differences in protein and lipid components in camel and bovine milk [34]. In this study, we first performed correlation analysis of 23 significantly altered metabolites and 17 differential proteins, which revealed strong associations between the two datasets. Further analysis revealed that the eight upregulated lipid metabolites were significantly correlated with nine upregulated proteins, and the 15 downregulated metabolites were significantly correlated with eight downregulated proteins, indicating a high consistency between the changes in metabolites and proteins in the regulation of milk composition. Moreover, Pearson correlation analysis identified 33 positive and 14 negative metabolite–protein pairs. Among them, CDH3 exhibited the most significant correlations. CDH3 is primarily located in the myoepithelial cells of the mammary gland. It can also be produced by the luminal epithelial cells during the lactation period, releasing soluble extracellular fragments. In colostrum, CDH3 mainly reflects mammary gland remodeling and barrier stability, while in mature milk, it is more associated with sustained lactation, epithelial homeostasis, and protein release [35]. Finally, Bovine Mammary Progenitor Cells are positively associated with 10 and negatively associated with five metabolites, suggesting its potential role in the dynamic regulation of milk composition [36]. Meanwhile, SM (20:0;2O/44:11) was significantly correlated with six proteins, suggesting its importance in both lipid metabolism and regulation of milk proteins [37]. SM (20:0;2O/44:11) participates in the construction of the milk fat globule membrane. In colostrum, it contributes to intestinal barrier, immunity, and anti-infection. In mature milk, it supports the development of the intestinal tract, nervous system, and myelin sheath, and reflects the remodeling of lipid metabolism during the lactation period [38]. O2PLS analysis further identified TG (10:0_11:0_12:0) and CSN1S1 as potential core biomarkers of the differences between colostrum and mature milk [6,39]. TG (10:0_11:0_12:0) is a triacylglycerol containing medium-chain fatty acids in milk, which is located in the core of milk fat globules. In colostrum, it primarily provides early energy and aids in intestinal defense. In mature milk, its abundance generally increases, continuously supporting fat absorption, energy supply, and growth and development [40]. The αS1-casein encoded by CSN1S1 has a relatively low content in colostrum, primarily providing early-stage proteins and minerals. Its content increases in mature milk, continuously supporting nutritional supply, growth, and development, and reflecting the maturity of mammary gland secretion [41].

Finally, we conducted a joint analysis of 933 differentially expressed proteins and 945 differentially expressed metabolites based on KEGG pathways, and a total of 13 pathways were enriched. Among these, metabolic pathways and glycerophospholipid metabolism were the most significantly enriched, which is consistent with the results of the differential metabolite and protein analyses described above. These research findings not only clarify the systemic interactions between metabolites and proteins, but also provide key molecular evidence for revealing the differences in nutritional components and functional properties between colostrum and mature milk.

Finally, the focus was given to the α-Ketoglutarate (α-KG) which presents in both colostrum and mature milk. α-KG is a key intermediate of the tricarboxylic acid (TCA) cycle, playing a central role in energy metabolism, amino acid biosynthesis and signaling pathways [42]. We found that the α-KG level was significantly lower in colostrum than that in mature milk, suggesting its crucial metabolic regulatory role during milk maturation [43].

The correlation analysis between α-KG and 14 differential metabolites enriched in 13 KEGG pathways showed significant correlations between α-KG and several phospholipids, including LPC (20:4), PS (40:5), PC (38:4), and LPC (17:0). This correlation suggests α-KG may influence milk composition through membrane lipid metabolism and signaling lipid pathways [44]. Additionally, α-KG was significantly correlated with key enzymes of the TCA cycle, including IDH1, IDH2 and ACO1, suggesting a close relationship between α-KG levels and the abundance of TCA cycle enzymes. Collectively, these findings indicate that α-KG is a key metabolite involved in the transition from colostrum to mature milk and plays an essential role in regulating the nutritional and functional characteristics of bovine milk [45].

The differential metabolites and proteins further highlighted the coordinated remodeling of milk lipid metabolism and mammary secretory function during the transition from colostrum to mature milk. TG, as the predominant lipid class in milk, reflects the capacity of the mammary gland to synthesize and secrete energy-rich milk fat, whereas SM is an important component of the milk fat globule membrane and contributes to membrane integrity and bioactive signaling. The differential abundance of CDH3, a cell-adhesion protein associated with mammary epithelial organization, may indicate changes in the cellular and structural state of the mammary gland during lactation establishment. Meanwhile, CSN1S1, a major casein protein involved in nutrient supply and casein micelle formation, reflects the maturation of milk protein secretion. In addition, the lower α-KG level in colostrum may indicate altered carbon–nitrogen metabolism and metabolic redistribution during the establishment of lactation. Collectively, these changes suggest that the transition from colostrum to mature milk involves coordinated remodeling of lipid metabolism, milk fat globule membrane organization, mammary epithelial status, and protein secretion rather than isolated changes in individual milk components.

This study has several limitations. First, the relatively small sample size of eight cows may limit the statistical power and generalizability of the findings, although the paired design reduced inter-animal variation. Second, the lipidomic, proteomic, and correlation analyses were exploratory and did not establish causal relationships. Further studies with larger cohorts and functional validation are needed to confirm these findings.

## 5. Conclusions

This study demonstrates that the transition from colostrum to mature milk in primiparous Holstein cows involves coordinated remodeling of lipid, protein, and central energy metabolism. Integrated lipidomic and proteomic analyses revealed that the coordinated associations among TG, sphingomyelin, CDH3, CSN1S1, and other differential molecules further indicate functional interactions between lipid and protein metabolism during milk maturation. The lower α-ketoglutarate abundance in colostrum and its associations with phospholipids and TCA cycle-related proteins further suggest altered central carbon metabolism. Overall, milk maturation is characterized by an integrated reorganization of lipid metabolism, protein synthesis/secretion, and energy metabolism, contributing to the distinct nutritional and functional properties of colostrum and mature milk.

## Data Availability

The original contributions presented in the study are included in the article, and further inquiries can be directed to the corresponding authors. The mass spectrometry proteomics data have been deposited to the ProteomeXchange Consortium via the PRIDE partner repository with the dataset identifier PXD083141.
